# Exogenous Nucleotides as Functional Food Supplements: A Bibliometric Analysis of Global Research Trends (2000–2025)

**DOI:** 10.3390/foods15122190

**Published:** 2026-06-17

**Authors:** Lunrongyi Tian, Meihong Xu

**Affiliations:** 1 The School of Public Health, Peking University, Beijing 100191, China; 2210306224@stu.pku.edu.cn; 2 Department of Nutrition and Food Hygiene, School of Public Health, Beijing Key Laboratory of Toxicological Research and Risk Assessment for Food Safety, Peking University, Beijing 100191, China; 3 Institute of Medical Technology, Peking University Health Science Center, Beijing 100019, China

**Keywords:** exogenous nucleotides, bibliometric analysis, dietary supplementation, healthy aging, aquaculture

## Abstract

**Background:** Exogenous nucleotides are bioactive compounds involved in nucleic acid synthesis, cellular metabolism, intestinal function, immune regulation, and related physiological processes. Owing to their potential roles in supporting growth, gut health, immune function, metabolic regulation, and physiological resilience, they have attracted increasing attention as functional dietary supplements and feed additives. However, the global research landscape of exogenous nucleotides has not been systematically characterized. This study aimed to map the development of this field and identify its major contributors, knowledge structures, application domains, and emerging research hotspots. **Methods:** Global literature on exogenous nucleotides published between 2000 and 2025 was retrieved from the Web of Science Core Collection. After screening and data standardization, 710 records were analyzed using VOSviewer, CiteSpace, and R-based visualization tools. The bibliometric analysis included publication output, country and institutional collaboration, keyword co-occurrence, co-cited references and journals, and citation burst detection. **Results:** A total of 710 publications were included. Annual publication output showed an overall upward trend, with marked growth after 2017. China and the United States were the leading contributors, while the Chinese Academy of Sciences and Peking University were among the most productive institutions. Keyword and co-citation analyses identified three major research themes: basic molecular mechanisms, physiological and health-related effects, and practical applications. Aquaculture and animal nutrition represented the most prominent application areas, with studies mainly focusing on growth performance, feed utilization, intestinal morphology, immune responses, oxidative stress resistance, and disease resistance. In human nutrition, research was mainly related to infant nutrition, intestinal and immune health, nutritional recovery, metabolic regulation, and healthy aging. Burst detection indicated a shift in research attention from early topics such as human milk and receptors to intestinal morphology and, more recently, nicotinamide mononucleotide and molecular activation. **Conclusions:** Research on exogenous nucleotides has expanded rapidly and is moving toward more mechanism-oriented and more diverse applications. Current evidence suggests that exogenous nucleotides have potential value as functional dietary supplements and feed additives, particularly in aquaculture, animal nutrition, infant nutrition, gut and immune health, metabolic regulation, and healthy aging. Future studies should further clarify compound-specific mechanisms, effective dose ranges, bioavailability, long-term safety, and population- or species-specific benefits to support their evidence-based application in functional foods, dietary supplements, infant formula, clinical nutrition, and functional feed products.

## 1. Introduction

Nucleotides are bioactive compounds that are essential for nucleic acid synthesis and are involved in a wide range of metabolic and regulatory processes, including energy metabolism, intestinal function, immune responses, and cellular signaling [1,2,3]. In addition to endogenous de novo and salvage pathways, nucleotides can also be supplied exogenously through foods, breast milk, infant formula, enteral nutrition products, dietary supplements, and animal feeds [4,5,6,7]. In this context, exogenous nucleotides broadly refer to dietary or supplemental nucleotides, nucleosides, and related nucleotide-derived compounds that enter the body from external sources. Natural sources include human milk, milk-based products, meat, fish, seafood, organ tissues, legumes, mushrooms, and yeast-derived ingredients, especially nucleic-acid-rich materials such as yeast RNA and yeast extracts.

Although nucleotides can be synthesized endogenously, exogenous supply may become nutritionally relevant under conditions of rapid growth, intestinal development, immune activation, infection, stress, tissue repair, aging, or limited endogenous synthesis [8,9,10]. Tissues with high rates of proliferation or metabolic activity, such as intestinal epithelial cells and immune cells, may have increased demand for nucleotide precursors. Therefore, exogenous nucleotides have been regarded as conditionally essential or functional dietary components with potential roles in supporting intestinal integrity, immune function, cellular metabolism, growth, recovery, and physiological resilience [11,12,13].

Accumulating evidence suggests that exogenous nucleotides may exert beneficial effects in both human and animal nutrition. In early-life and pediatric nutrition, exogenous nucleotides have been studied in relation to infant formula, intestinal maturation, immune development, and nutritional recovery [14,15,16]. In clinical and aging-related contexts, nucleotide-related compounds such as uridine, nicotinamide riboside, nicotinamide mononucleotide, and other NAD^+^-related precursors have attracted interest because of their potential roles in inflammation, mitochondrial function, metabolic regulation, and healthy aging [17,18,19,20]. In animal nutrition, especially aquaculture and livestock production, dietary nucleotides and yeast-derived nucleotide preparations have been investigated as functional feed additives for improving growth performance, feed utilization, intestinal morphology, immune responses, stress tolerance, and disease resistance [21,22,23].

These application scenarios are directly relevant to food science and functional nutrition because exogenous nucleotides are not only naturally occurring bioactive components in foods but also potential functional ingredients for the development of dietary supplements, infant formula, enteral nutrition products, and functional feed additives [24,25,26]. In recent years, interest in exogenous nucleotides has expanded in parallel with growing interest in functional foods, precision nutrition, nutrition-based strategies for healthy aging, and sustainable animal production. At the same time, the demand for improved gut health, immune support, metabolic regulation, and reduced antibiotic use in animal production has stimulated research on nucleotides as functional nutritional ingredients and feed additives [27,28,29].

Despite this expanding body of literature, research on exogenous nucleotides remains widely distributed across food science, nutrition, biochemistry, immunology, gastrointestinal physiology, animal science, and aquaculture. Most previous studies have focused on specific nucleotide-related compounds, biological functions, target populations, or mechanistic pathways, whereas a global overview of publication trends, collaborative structures, knowledge bases, application domains, and emerging research hotspots is still lacking. A bibliometric analysis can help clarify how this field has developed over time, identify major contributors and thematic clusters, and reveal how basic nucleotide biology is being connected with practical applications in functional foods, dietary supplementation, infant nutrition, animal feeds, and food-related health promotion.

Accordingly, the present study outlines a bibliometric analysis of global research on exogenous nucleotides published between 2000 and 2025. Using Web of Science Core Collection data and knowledge-mapping tools, we analyzed publication output, country and institutional collaboration, core journals and references, and the evolution of research hotspots. This study aims to provide a structured overview of the field and to offer a useful reference for future research and evidence-based applications of exogenous nucleotides as functional food supplements and feed additives.

## 2. Materials and Methods

### 2.1. Data Source and Search Strategy

The bibliometric analysis was based on the Web of Science Core Collection (WoSCC), with records retrieved from the Science Citation Index Expanded (SCI-Expanded). WoSCC was selected because it provides standardized bibliographic and citation information, including titles, abstracts, keywords, author information, institutional affiliations, cited references, citation counts, and publication metadata. These data fields are required for mapping publication trends, co-authorship networks, institutional and country collaborations, keyword co-occurrence patterns, co-citation relationships, and the evolution of research frontiers.

WoSCC is widely used in bibliometric studies because of its curated indexing standards and relatively consistent citation records. Since the aim of this study was to characterize global research trends and knowledge structures rather than to conduct a comprehensive clinical systematic review, the use of a single standardized citation database was considered appropriate. This strategy also helped reduce duplicate records, inconsistencies in document classification, and variations in citation formats that may occur when merging records from multiple databases.

The search covered publications from 1 January 2000 to 31 December 2025. The search strategy was designed to identify studies related to exogenous nucleotides and their nutritional, dietary, supplemental, or feed-related applications. To improve topic specificity, nucleotide-related terms were searched in the title field, whereas terms related to exogenous intake, dietary supplementation, and nutritional application were searched in the topic field. All searches were completed on 31 December 2025. The full search strategy is shown in Box 1.

Box 1 **Search query used in the Web of Science Core Collection.**

TI = (“nucleotid” OR “ribonucleotid” OR “nucleoside*” OR “ribonucleoside*” OR “nucleic acid*” OR “5′-AMP” OR “5′-CMP” OR “5′-UMP” OR “5′-GMP” OR “5′-IMP” OR “cAMP” OR “cGMP” OR “NAD*” OR “NMN” OR “uridine” OR “adenosine” OR “cytidine” OR “yeast extract” OR “yeast RNA”)

AND

TS = (“diet” OR “exogenous” OR “supplement” OR “additiv*” OR “intake” OR “oral” OR “administra*” OR “formula” OR “feed” OR “nutrition*”)

Timespan: 2000-01-01 to 2025-12-31

### 2.2. Eligibility Criteria and Study Selection

Only articles and reviews published in English were eligible for inclusion. Conference abstracts, editorial materials, letters, corrections, book chapters, and other publication types lacking complete bibliographic information were excluded. After the initial search, records were screened according to document type, subject relevance, and topic relevance. Titles and abstracts were reviewed manually, and full texts were checked when necessary.

**Figure 1:**
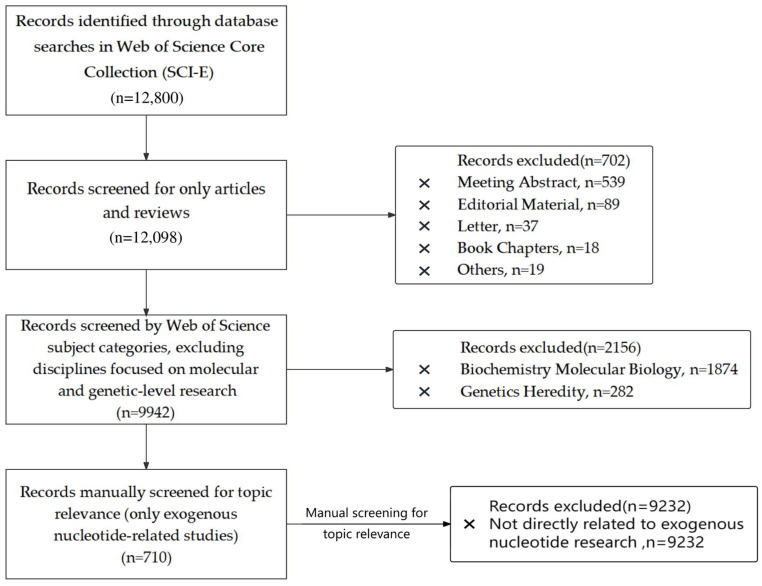
Flow diagram of the literature screening and bibliometric analysis. The symbol (×) indicates records excluded during the screening process.

Studies were included if they primarily addressed exogenous nucleotides, nucleosides, or nucleotide-related compounds in relation to dietary intake, nutritional supplementation, feed application, physiological regulation, or health-related outcomes. Studies were excluded if they focused predominantly on endogenous nucleotide biosynthesis, molecular or genetic mechanisms unrelated to exogenous intake, or if nucleotide-related terms appeared only incidentally without representing the main topic of the study. Duplicate records and records with incomplete bibliographic information were also removed. After screening, 710 publications were included in the final bibliometric analysis (Figure 1).

### 2.3. Data Extraction and Standardization

Full records and cited references of the included publications were downloaded from WoSCC in plain text format. Extracted information included authors, affiliations, countries, publication years, keywords, source journals, cited references, and citation counts. Before analysis, the dataset was standardized manually. This process included correction of incomplete or obviously erroneous records, unification of different name formats for the same author or institution, harmonization of keyword spelling variants, and merging of synonymous high-frequency keywords. Collaboration information at the country and institutional levels was also checked and standardized where necessary.

### 2.4. Bibliometric Analysis and Visualization

Bibliometric analysis and visualization were performed using CiteSpace (version 6.3.R1), VOSviewer (version 1.6.20), and R software (version 4.3.1). Annual publication output was summarized to describe temporal trends in research activity. CiteSpace was used to conduct citation burst detection and identify changes in research attention over time, with the minimum burst duration set to 1 year. VOSviewer was used to construct co-authorship, institutional collaboration, country collaboration, keyword co-occurrence, reference co-citation, and journal co-citation networks.

In the visualization maps, node size represented the relative frequency or weight of an item, link thickness indicated the strength of association, and colors represented clusters or temporal patterns generated by the software. R-based visualization packages, including ggplot2, treemapify, sf, rnaturalearth, ggrepel, and plotly, were used to generate supplementary visualizations, such as the country collaboration map and keyword frequency distribution. Final figures were adjusted only for readability and consistency of presentation.

Thresholds for network construction were determined according to the distribution of the dataset and the need to obtain interpretable maps. Specifically, authors with at least 5 publications, institutions with at least 6 publications, countries with at least 5 publications, keywords with at least 19 occurrences, co-cited references with at least 40 citations, and co-cited journals with at least 150 citations were included in the corresponding visual analyses.

## 3. Results

### 3.1. Annual Publication Output

**Figure 2:**
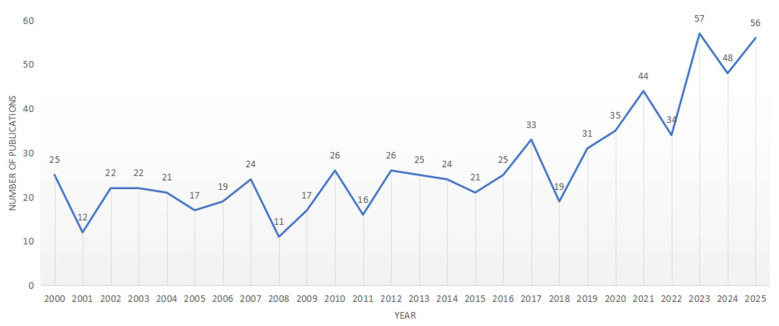
Annual publication output in exogenous nucleotide research, 2000–2025.

A total of 710 publications were included in this bibliometric analysis. Annual publication output showed an overall upward trend from 2000 to 2025. Between 2000 and 2016, the number of publications remained relatively stable, generally ranging from 10 to 30 papers per year, with the lowest output recorded in 2008 (11 papers). From 2017 onward, publication output increased more markedly, rising from 33 papers in 2017 to a peak of 57 papers in 2023. Although the number declined to 48 in 2024, it rebounded to 56 in 2025, indicating sustained research activity in this field (Figure 2).

### 3.2. Author Collaboration Analysis

A total of 3752 authors contributed to the literature on exogenous nucleotides. Table 1 presents the top 10 authors by publication output, whereas Table 2 presents the top 10 authors by total citation count. Among the most productive authors, Yong Li ranked first with 18 publications, followed by Meihong Xu with 17 publications, Mehmet Cansev with 16 publications, Angel Gil with 12 publications, and Xin Wu from the Chinese Academy of Sciences with 12 publications. In the citation-based ranking, Shin-Ichiro Imai ranked first, followed by Carles Canto, Johan Auwerx, Anthony A. Sauve, and Leonard Guarente.

**Figure 3:**
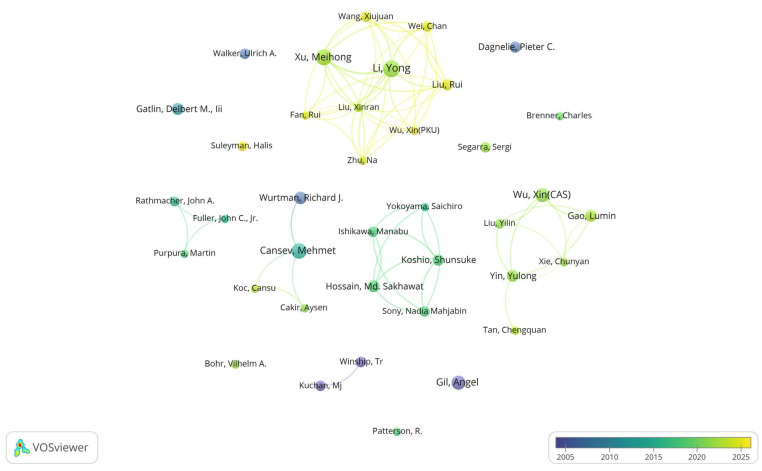
Author collaboration network in exogenous nucleotide research, 2000–2025.

The author collaboration network included 38 authors with at least five publications and showed several identifiable clusters (Figure 3). One major cluster was centered on Peking University, where Yong Li and Meihong Xu collaborated closely with Xin Wu, Liu Rui, Wang Xiujuan, and Wei Chan. Another cluster linked Mehmet Cansev and colleagues at Bursa Uludağ University with Richard J. Wurtman at the Massachusetts Institute of Technology. A third cluster was centered on Kagoshima University and was mainly related to aquaculture nutrition. Another Chinese Academy of Sciences-linked cluster focused on nucleotide-related nutrition and nicotinamide mononucleotide research in livestock. The network also contained several isolated nodes, indicating that author collaboration was unevenly distributed across the field.

### 3.3. Institutional Collaboration Analysis

A total of 1117 institutions were identified in the dataset. Table 3 presents the top 10 institutions by publication output, whereas Table 4 presents the top 10 institutions by total citation count. The Chinese Academy of Sciences ranked first in publication output with 30 publications, followed by Peking University with 21 publications, Abbott Laboratories with 18 publications, Bursa Uludağ Üniversitesi with 17 publications, and the University of Granada with 16 publications. In the citation-based ranking, the Massachusetts Institute of Technology ranked first, followed by the University of Washington, École Polytechnique Fédérale de Lausanne, Weill Cornell Medicine, and the University of Pennsylvania.

**Figure 4:**
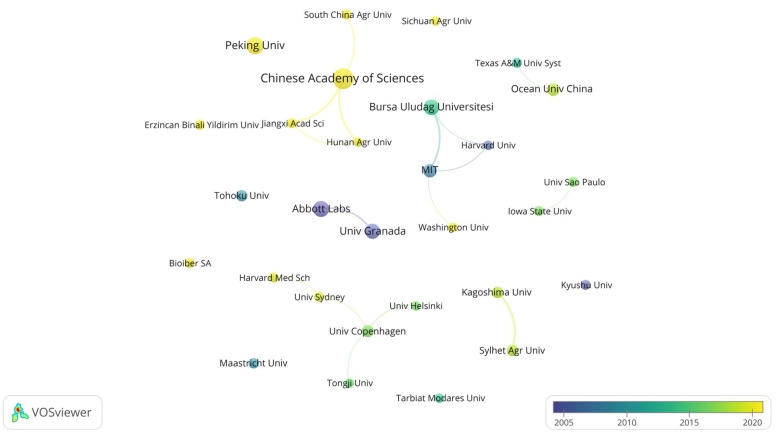
Institutional collaboration network in exogenous nucleotide research, 2000–2025. Note: Institution names in the VOSviewer map are displayed without Turkish diacritical marks. For example, “Bursa Uludag Universitesi” refers to “Bursa Uludağ Üniversitesi” (Bursa Uludağ University).

Among institutions with at least six publications, the collaboration network showed that the Chinese Academy of Sciences occupied the most prominent position, with strong links to several Chinese universities and research institutes (Figure 4). Bursa Uludağ Üniversitesi formed a cross-regional cluster with institutions in Europe and the United States. The University of Copenhagen served as the core connecting node of another major cluster. Overall, the institutional map showed participation from both academic and industrial organizations and reflected an internationally distributed research landscape.

### 3.4. Country Collaboration Analysis

**Figure 5:**
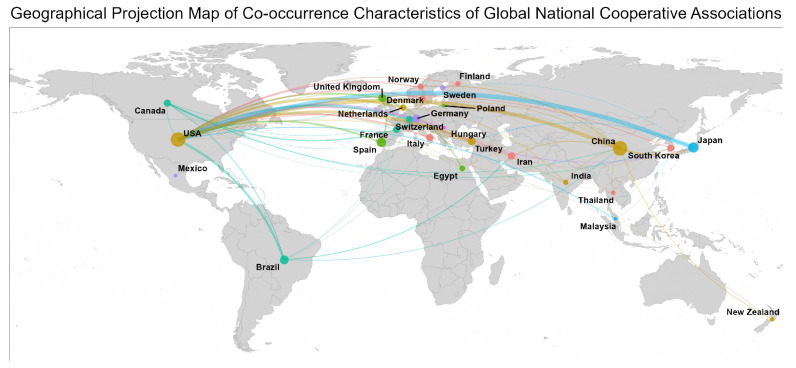
Country collaboration network in exogenous nucleotide research, 2000–2025. The colored dots represent different countries/regions, where the size of each dot corresponds to the total publication volume of the country; multicolored lines between dots indicate collaborative relationships between nations, and the thickness of lines reflects the cooperation frequency between two countries.

Research on exogenous nucleotides involved 60 countries. The United States (175 publications) and China (169) were the two leading contributors, followed by Japan (67), Spain (53), Brazil (44), Germany (35), and England (30) (Table 5). In the collaboration network constructed from countries with at least five publications, the United States and China appeared as the largest and most connected nodes (Figure 5). England, Germany, Japan, and France also occupied important intermediary positions. The overlay visualization suggested more recent activity in countries such as China, Turkey, and Brazil, whereas countries such as Germany and Switzerland appeared earlier in the field. These results indicate a globally distributed but unequally concentrated pattern of research activity.

### 3.5. Keyword Co-Occurrence Analysis

**Figure 6:**
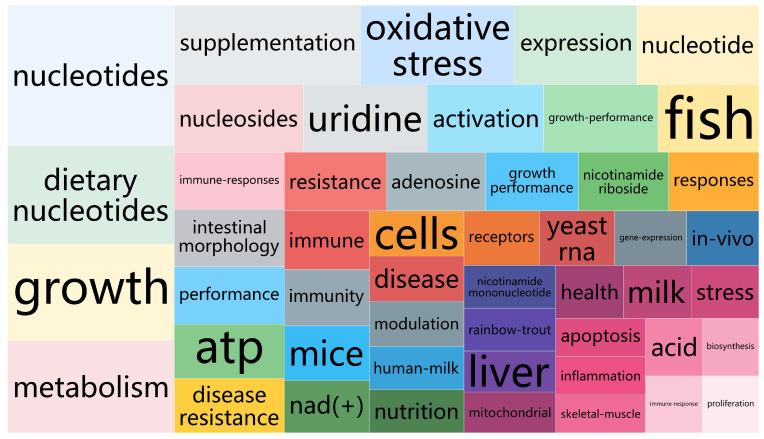
Keyword frequency distribution in exogenous nucleotide research, 2000–2025. The area of each rectangle is positively proportional to the occurrence frequency of the corresponding keyword; a larger rectangle means a higher frequency. Colors are only used to distinguish different keywords.

**Figure 7:**
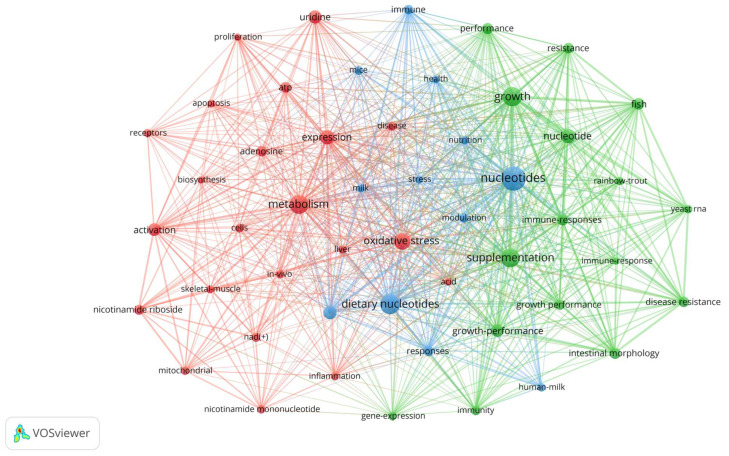
Keyword co-occurrence network and cluster structure in exogenous nucleotide research, 2000–2025. Distinct colors denote separate clusters, while node size reflects keyword occurrence frequency.

A total of 3917 keywords were identified, of which 50 with at least 19 occurrences were included in the co-occurrence analysis. The most frequent terms were nucleotides and dietary nucleotides, followed by keywords such as metabolism, growth, performance, oxidative stress, and fish (Figure 6). The keyword network formed three major clusters (Figure 7). The first cluster was centered on oxidative stress, metabolism, and gene expression and was associated with molecules such as uridine, NAD^+^, NMN, and nicotinamide riboside. The second cluster focused on dietary nucleotides, nucleosides, immunity, and cells, indicating research on physiological and health-related effects. The third cluster was centered on growth, immune responses, supplementation, fish, disease resistance, and intestinal morphology, highlighting practical applications, particularly in aquaculture. Together, these clusters indicate that the field is organized around three major themes: basic molecular mechanisms, physiological and health effects, and application-oriented research.

### 3.6. Co-Cited Reference Analysis

**Figure 8:**
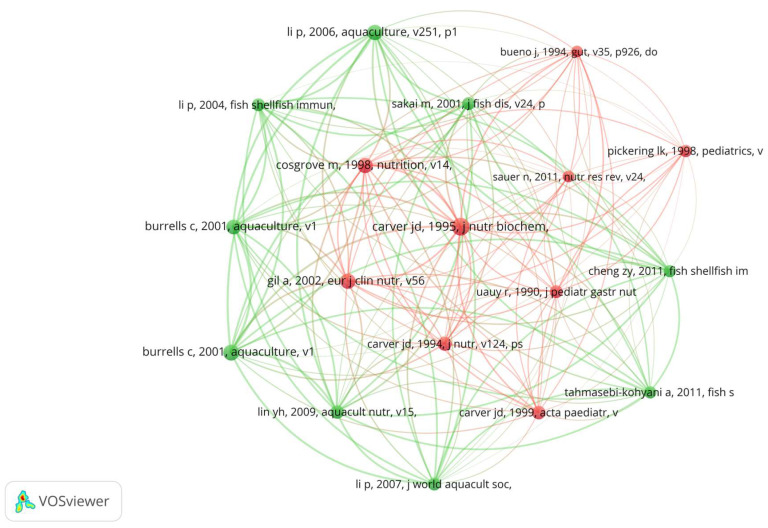
Co-cited reference network and cluster structure in exogenous nucleotide research, 2000–2025. Different colors represent distinct clusters, and the size of each node corresponds to the co-citation count of the reference.

A total of 24,123 co-cited references were identified. The most frequently co-cited article was Carver JD (1995) [30], with 100 co-citations, followed by Burrells C (2001) [31], with 79, and Gil A (2002) [32], with 75 (Table 6). Other highly co-cited references included Cosgrove M (1998) [33] and Li P (2006) [34], each with 70 co-citations. In the co-citation network, one prominent cluster focused on aquaculture and fish immunity, centered on studies by Li P and Burrells C (Figure 8). Another major cluster focused on nutrition and pediatric medicine, centered on Carver JD and linked to early foundational studies in infant nutrition, immunity, and gastrointestinal development. These co-cited references represent the principal intellectual foundations of exogenous nucleotide research.

### 3.7. Co-Cited Journal Analysis

**Figure 9:**
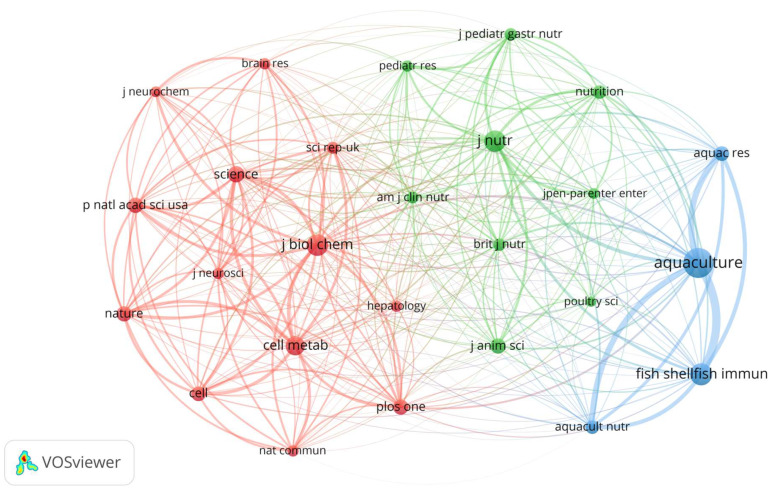
Co-cited journal network and cluster structure in exogenous nucleotide research, 2000–2025. Different colors represent distinct clusters, and the size of each node corresponds to the co-citation frequency of the journal.

A total of 4978 co-cited journals were identified. *Aquaculture* was the most highly co-cited journal (1174 co-citations), followed by *Fish & Shellfish Immunology* (648), *Journal of Biological Chemistry* (627), *Journal of Nutrition* (622), and *Cell Metabolism* (497) (Table 7). The journal co-citation network formed three major clusters (Figure 9). One cluster consisted mainly of basic life science and high-impact multidisciplinary journals, including *Nature*, *Science*, and *Cell*. A second cluster was composed of nutrition and medical journals, including *Journal of Nutrition* and *American Journal of Clinical Nutrition*. The third cluster consisted primarily of aquaculture and fish immunology journals, centered on *Aquaculture* and *Fish & Shellfish Immunology*. The network structure suggests that exogenous nucleotide research is supported by knowledge bases from basic life sciences, nutrition and medicine, and aquaculture-oriented application research.

### 3.8. Burst Detection Analysis

**Figure 10:**
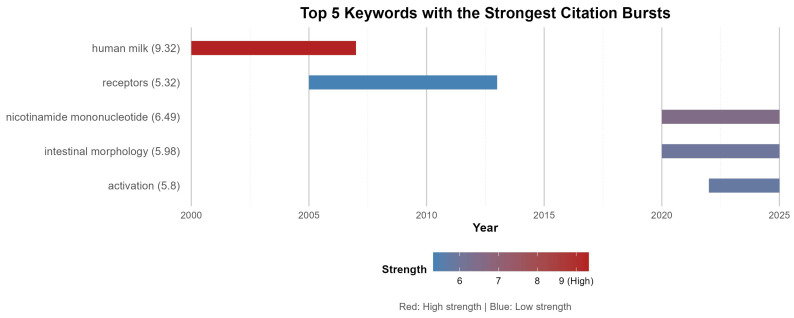
Citation burst detection of research hotspots in exogenous nucleotide research, 2000–2025.

Burst detection analysis revealed clear temporal changes in research focus (Figure 10). In the early stage, human milk showed the strongest burst from 2000 to 2007, with a burst strength of 9.32, while receptors showed a burst from 2005 to 2013, with a burst strength of 5.32. In the intermediate stage, intestinal morphology emerged as a major burst term from 2016 to 2020, with a burst strength of 5.98. In the most recent stage, nicotinamide mononucleotide showed a burst from 2020 to 2025, with a burst strength of 6.49, and activation showed a burst from 2022 to 2025, with a burst strength of 5.80. Overall, the burst pattern showed a shift in research attention from early topics related to human milk and receptor-related signaling to intestinal morphology and gut-related applications, and more recently to nicotinamide mononucleotide and mechanism-oriented research.

## 4. Discussion

This bibliometric analysis provides a global overview of research on exogenous nucleotides as functional food supplements from 2000 to 2025. The results show that the field has experienced a phased development pattern, with relatively stable publication output before 2017 and a marked increase thereafter. The increased publication activity after 2017 may have occurred in parallel with growing interest in functional foods, precision nutrition, healthy aging, sustainable animal production, and nutritional strategies related to intestinal health, immune function, and metabolic regulation [40,41,42,43,44].

The country and institutional analyses indicate that research activity in this field is globally distributed but unevenly concentrated. The United States and China were the two leading contributors, while Japan, Spain, Brazil, Germany, England, Australia, Iran, and Italy also made notable contributions. The United States has contributed substantially to foundational research on nucleotide metabolism, NAD^+^ biology, infant nutrition, and immune-related functions, whereas China has become increasingly active in recent years, particularly in nutrition, animal science, aquaculture, and nucleotide-related aging research [36,45,46,47,48].

The author and institutional analyses also show that publication productivity and citation impact do not fully overlap. Some authors and institutions produced many publications but had relatively recent average publication years, whereas several highly cited contributors published fewer but highly influential papers. This distinction suggests that productivity, citation impact, and research maturity should be considered together when evaluating the development of this field.

The collaboration networks further show that exogenous nucleotide research has formed several recognizable academic clusters. These include a nutrition and aging-related cluster centered on Peking University, a neurobiology and nucleotide metabolism-related cluster involving Bursa Uludağ University and the Massachusetts Institute of Technology, and aquaculture-oriented clusters involving institutions in Japan, China, and other countries. The participation of both academic institutions and industry-related organizations, such as Abbott Laboratories, also indicates that the field has practical relevance for nutrition products, infant formula, functional foods, and feed additives [49,50]. However, the presence of multiple isolated nodes suggests that collaboration remains fragmented. Stronger international and interdisciplinary collaboration may help connect basic nucleotide biology with translational research in food science, human nutrition, animal nutrition, and aquaculture.

Keyword co-occurrence, co-cited reference, and co-cited journal analyses indicate that the knowledge structure of this field can be broadly organized around three interconnected dimensions: molecular mechanisms, physiological and health-related effects, and practical applications. Mechanistic research mainly involves oxidative stress, metabolism, gene expression, mitochondrial function, inflammatory regulation, and NAD^+^-dependent pathways. Health-related research focuses on intestinal development, gut barrier function, immune modulation, inflammation, microbiota-related effects, nutritional recovery, and aging-related outcomes. Application-oriented research is most prominent in aquaculture and animal nutrition, where dietary nucleotides and yeast-derived nucleotide preparations have been studied as functional feed additives to improve growth performance, feed utilization, intestinal morphology, immune responses, stress tolerance, oxidative stress resistance, and disease resistance [51,52,53,54,55].

Among the different application areas, aquaculture appears to be the most mature and visible field for exogenous nucleotide research. This is supported by the high frequency of keywords related to fish, growth, immune responses, supplementation, disease resistance, and intestinal morphology, as well as by the strong co-citation influence of journals such as *Aquaculture* and *Fish & Shellfish Immunology*. In aquaculture, exogenous nucleotides may be especially valuable because aquatic animals are frequently exposed to intensive farming conditions, pathogen pressure, dietary changes, and stress. Under these conditions, exogenous nucleotides may support intestinal integrity, immune competence, growth performance, and resilience [56,57,58]. These findings suggest that functional feed additives remain a major translational pathway for exogenous nucleotide research.

In livestock and poultry production, dietary nucleotides have also been investigated for their potential to support intestinal barrier function, immune regulation, and resistance to pathogen challenge [59,60]. This application is relevant to the development of antibiotic-reduction strategies in animal production. Yeast-derived nucleotides, yeast RNA, and nucleotide-enriched diets have been studied as possible alternatives or complements to traditional feed additives, particularly in young or stressed animals [61,62,63]. However, more standardized studies are still needed to clarify optimal nucleotide sources, supplementation levels, duration of intervention, and species-specific responses.

In human nutrition, exogenous nucleotides have been studied mainly in relation to infant formula, pediatric nutrition, nutritional recovery, gut health, immune support, metabolic regulation, and healthy aging [64,65]. Early research focused strongly on human milk, infant nutrition, and immune-related functions, reflecting the biological importance of nucleotides in growth and intestinal development [36,66]. More recent studies have expanded toward nucleotide-related compounds such as uridine, nicotinamide riboside, nicotinamide mononucleotide, and other NAD^+^-related precursors [67,68]. This shift suggests that the field is moving from broad dietary exogenous nucleotides toward more compound-specific and mechanism-driven investigation.

The potential health value of exogenous nucleotides may be explained by their biological roles in tissues with high metabolic or proliferative demand. Intestinal epithelial cells, immune cells, and other rapidly renewing tissues require sufficient nucleotide precursors for nucleic acid synthesis, cellular repair, and functional maintenance [69,70]. Under conditions such as rapid growth, infection, inflammation, stress, malnutrition, or aging, exogenous nucleotide supply may help reduce the metabolic cost of de novo synthesis and support salvage pathways [71,72,73]. In addition, nucleotide-related compounds may influence immune and inflammatory signaling, oxidative stress, mitochondrial function, gut microbiota composition, and NAD^+^-dependent metabolic regulation [58,74,75]. Therefore, the functional effects of exogenous nucleotides are likely to involve multiple interconnected pathways rather than a single mechanism.

The burst detection results further reflect the evolution of research priorities. Early burst terms such as human milk and receptors indicate initial interest in early-life nutrition and signaling-related functions. The later emergence of intestinal morphology reflects growing attention to gut structure, barrier function, and feed-related applications. More recently, nicotinamide mononucleotide and activation have become prominent burst terms, suggesting increasing interest in NAD^+^ metabolism, cellular signaling, metabolic regulation, and healthy aging. This evolution indicates that future research is likely to place greater emphasis on compound-specific mechanisms, dose–response relationships, bioavailability, safety, and translational relevance.

Overall, the scientific value of this bibliometric analysis lies in connecting a fragmented body of literature across food science, nutrition, biochemistry, immunology, animal science, and aquaculture. Rather than showing only that publication output has increased, the present analysis identifies how the field has evolved from early studies on human milk, infant nutrition, and immune function to current interests in aquaculture applications, gut health, NAD^+^-related metabolism, and healthy aging. It also highlights the main translational directions of exogenous nucleotides as functional dietary supplements and feed additives. These findings may help researchers and product developers identify priority areas, including infant nutrition, intestinal and immune health, healthy aging, sustainable animal production, and functional feed formulation.

Despite this progress, several gaps remain. First, much of the current evidence is derived from animal models, aquaculture studies, or feed-related applications, whereas high-quality human intervention studies remain relatively limited. Second, different nucleotide sources, including purified nucleotides, nucleosides, yeast-derived preparations, and NAD^+^ precursors, may differ in bioavailability, metabolism, and biological effects. Third, effective dose ranges, supplementation duration, long-term safety, and population- or species-specific benefits remain insufficiently defined. Fourth, outcome measures are heterogeneous across studies, making it difficult to compare findings or establish standardized recommendations. Future research should therefore combine well-designed human trials, production-based animal studies, multi-omics analysis, gut microbiota profiling, and molecular pathway validation to better connect mechanistic evidence with practical applications.

This study also has limitations. First, the bibliometric dataset was obtained from the Web of Science Core Collection, specifically the SCI-Expanded index. Although WoSCC provides standardized citation records and is well suited for bibliometric mapping, publications indexed exclusively in other databases, such as Scopus, PubMed, Embase, Google Scholar, regional databases, or patent databases, may not have been fully captured. Therefore, the findings should be interpreted as representing the WoSCC/SCI-Expanded-indexed literature on exogenous nucleotides as functional food supplements. Second, the results may be influenced by the search strategy, field selection, and data-standardization procedures. In this study, nucleotide-related terms were searched in the title field to improve topic specificity and reduce irrelevant records. While this strategy enhanced dataset relevance, it may have excluded studies in which nucleotide-related terms appeared only in abstracts or keywords. The broad search strategy may also have influenced the observed clusters and publication trends. Since the dataset included nucleotides, nucleosides, nucleic acid-related materials, yeast-derived nucleotide sources, and NAD^+^-related compounds, the identified clusters reflect a heterogeneous but nutritionally related field rather than a single chemically uniform category. Therefore, trends such as the increase in publications should be interpreted as growth in broader exogenous nucleotide-related research, not necessarily as a uniform increase in studies on classical dietary nucleotides alone. Despite these limitations, the long study period from 2000 to 2025 and the combined use of VOSviewer, CiteSpace, and R-based visualization tools provide a reproducible overview of publication trends, collaboration patterns, knowledge structures, and emerging hotspots in this field.

## 5. Conclusions

This bibliometric analysis provides a systematic overview of global research on exogenous nucleotides as functional food supplements from 2000 to 2025. The field has grown markedly, particularly after 2017, and is mainly organized around three interconnected directions: basic molecular mechanisms, physiological and health-related effects, and practical applications. Aquaculture and animal nutrition represent the most mature application areas, while infant nutrition, intestinal and immune health, metabolic regulation, and healthy aging are important directions in human nutrition.

The findings indicate that exogenous nucleotides have potential value as functional dietary supplements and feed additives, with research interest gradually shifting from general exogenous nucleotides toward compound-specific and mechanism-oriented studies involving uridine, NAD^+^-related compounds, and nicotinamide mononucleotide. Future research should further clarify effective dose ranges, bioavailability, long-term safety, mechanisms of action, and population- or species-specific benefits to support their evidence-based application in functional foods, dietary supplements, infant formula, clinical nutrition, and functional feed products.

## Figures and Tables

**Table 1:** Top 10 authors by publication output in exogenous nucleotide research.

Author	Documents	Citations	Average Citations	Average Publication Year ^2^
Li, Yong	18	247	13.7	2021.2
Xu, Meihong	17	223	13.1	2021.6
Cansev, Mehmet	16	752	47.0	2013.2
Gil, Angel	12	443	36.9	2003.6
Wu, Xin (CAS) ^1^	12	216	18.0	2020.9
Wurtman, Richard J.	10	749	74.9	2007.1
Gatlin, Delbert M., III	9	737	81.9	2011.1
Hossain, Md. Sakhawat	9	340	37.8	2017.9
Koshio, Shunsuke	9	340	37.8	2017.9
Gao, Lumin	9	156	17.3	2021.8

^1^ (CAS) indicates the author Xin Wu is affiliated with the Chinese Academy of Sciences (CAS). ^2^ Average publication year, calculated as the arithmetic mean of the publication years of an author’s included documents.

**Table 2:** Top 10 authors by citations in exogenous nucleotide research.

Author	Documents	Citations	Average Citations	Average Publication Year ^1^
Imai, Shin-ichiro	2	1843	921.5	2016.0
Canto, Carles	3	1233	411.0	2017.7
Auwerx, Johan	2	1232	616.0	2014.0
Sauve, Anthony A.	2	1232	616.0	2014.0
Guarente, Leonard	2	1179	589.5	2016.5
Baur, Joseph A.	4	1154	288.5	2021.0
Pirinen, Eija	2	1041	520.5	2017.5
Houtkooper, Riekelt H.	3	1024	341.3	2019.3
Andreux, Penelope A.	1	1001	1001.0	2012.0
Cen, Yana	1	1001	1001.0	2012.0

^1^ Average publication year, calculated as the arithmetic mean of the publication years of an author’s included documents.

**Table 3:** Top 10 institutions by publication output in exogenous nucleotide research.

Organization	Documents	Citations	Average Citations	Average Publication Year ^1^
Chinese Academy of Sciences	30	560	18.7	2020.4
Peking University	21	263	12.5	2020.5
Abbott Laboratories	18	507	28.2	2002.9
Bursa Uludağ Üniversitesi	17	775	45.6	2013.5
University of Granada	16	490	30.6	2005.7
Massachusetts Institute of Technology	13	1943	149.5	2008.0
Ocean University of China	11	470	42.7	2018.0
University of Copenhagen	10	993	99.3	2016.3
University of New South Wales	10	991	99.1	2017.2
São Paulo State University	10	99	9.9	2016.3

^1^ Average publication year, calculated as the arithmetic mean of the publication years of an institution’s included documents.

**Table 4:** Top 10 institutions by citations in exogenous nucleotide research.

Organization	Documents	Citations	Average Citations	Average Publication Year ^1^
Massachusetts Institute of Technology	13	1943	149.5	2008.0
Washington University	6	1875	312.5	2020.7
École Polytechnique Fédérale de Lausanne	4	1407	351.8	2018.0
Weill Cornell Medicine	3	1258	419.3	2011.3
University of Pennsylvania	5	1157	231.4	2021.8
University of Basel	4	1066	266.5	2010.0
University of Eastern Finland	1	1001	1001.0	2012.0
University of Copenhagen	10	993	99.3	2016.3
University of New South Wales	10	991	99.1	2017.2
National Institute on Aging	4	871	217.8	2020.8

^1^ Average publication year, calculated as the arithmetic mean of the publication years of an institution’s included documents.

**Table 5:** Top 10 countries by publication output in exogenous nucleotide research.

Country	Documents	Country	Documents
United States	175	Germany	35
China	169	England	30
Japan	67	Australia	28
Spain	53	Iran	27
Brazil	44	Italy	27

**Table 6:** Top 10 co-cited references in exogenous nucleotide research.

No.	Reference	Co-Citation Count
1	[30] Carver JD, 1995, *Journal of Nutritional Biochemistry*, v6, p58, doi 10.1016/0955-2863(94)00019-i	100
2	[31] Burrells C, 2001, *Aquaculture*, v199, p171, doi 10.1016/s0044-8486(01)00576-2	79
3	[32] Gil A, 2002, *European Journal of Clinical Nutrition*, v56, ps1, doi 10.1038/sj.ejcn.1601475	75
4	[33] Cosgrove M, 1998, *Nutrition*, v14, p748, doi 10.1016/s0899-9007(98)00075-6	70
5	[34] Li P, 2006, *Aquaculture*, v251, p141, doi 10.1016/j.aquaculture.2005.01.009	70
6	[35] Burrells C, 2001, *Aquaculture*, v199, p159, doi 10.1016/s0044-8486(01)00577-4	67
7	[36] Carver JD, 1994, *The Journal of Nutrition*, v124, ps144, doi 10.1093/jn/124.suppl_1.144s	61
8	[37] Carver JD, 1999, *Acta Paediatrica*, v88, p83, doi:10.1111/j.1651-2227.1999.tb01306.x	59
9	[38] Lin YH, 2009, *Aquaculture Nutrition*, v15, p117, doi 10.1111/j.1365-2095.2007.00561.x	59
10	[39] Li P, 2004, *Fish & Shellfish Immunology*, v16, p561, doi 10.1016/j.fsi.2003.09.005	54

**Table 7:** Top 10 co-cited journals in exogenous nucleotide research.

Journal	Citations
*Aquaculture*	1174
*Fish & Shellfish Immunology*	648
*Journal of Biological Chemistry*	627
*Journal of Nutrition*	622
*Cell Metabolism*	497
*Science*	362
*Proceedings of the National Academy of Sciences of the United States of America*	324
*Journal of Animal Science*	323
*Nature*	314
*PlOS ONE*	314

## Data Availability

No new data were created or analyzed in this study.
